# A Collaborative Quality Improvement Model and Electronic Community of Practice to Support Sepsis Management in Emergency Departments: Investigating Care Harmonization for Provincial Knowledge Translation

**DOI:** 10.2196/resprot.1597

**Published:** 2012-07-12

**Authors:** Kendall Ho, Julian Marsden, Sandra Jarvis-Selinger, Helen Novak Lauscher, Noreen Kamal, Rob Stenstrom, David Sweet, Ran D Goldman, Grant Innes

**Affiliations:** 1 eHealth Strategy OfficeFaculty of MedicineUniversity of British ColumbiaVancouver, BCCanada; 2 Department of Emergency MedicineFaculty of MedicineUniversity of British ColumbiaVancouver, BCCanada; 3 Stroke Services B.C.Provincial Health Services AuthorityVancouver, BCCanada; 4 Emergency and Critical Care MedicineVancouver General HospitalVancouver, BCCanada; 5 Department of PediatricsUniversity of British ColumbiaVancouver, BCCanada; 6 Division of Continuing Professional DevelopmentUniversity of British ColumbiaVancouver, BCCanada; 7 Emergency DepartmentFoothills Medical CentreCalgary, ABCanada

**Keywords:** Knowledge translation, continuous quality improvement, emergency medicine, sepsis

## Abstract

Emergency medicine departments within several organizations are now advocating the adoption of early intervention guidelines for patients with the signs and symptoms of sepsis. This proposed research will lead to a comprehensive understanding of how diverse emergency department (ED) sites across British Columbia (BC), Canada, engage in a quality improvement collaborative to lead to improvements in time-based process measures and clinical outcomes for septic patients in EDs. To address the challenge of sepsis management, in 2007, the BC Ministry of Health began working with emergency health professionals, including health administrators, to establish a provincial ED collaborative: Evidence to Excellence (E2E). The E2E initiative employs the Institute for Healthcare Improvement (IHI) model and is supported by a Web-based community of practice (CoP) in emergency medicine. It aims to (1) support clinicians in accessing and applying evidence to clinical practice in emergency medicine, (2) support system change and clinical process improvement, and (3) develop resources and strategies to facilitate knowledge translation and process improvement. Improving sepsis management is one of the central foci of the E2E initiative. The primary purpose of our research is to investigate whether the application of sepsis management protocols leads to improved time-based process measures and clinical outcomes for patients presenting to EDs with sepsis. Also, we seek to investigate the implementation of sepsis protocols among different EDs. For example: (1) How can sepsis protocols be harmonized among different EDs? (2) What are health professionals’ perspectives on interprofessional collaboration with various EDs? and (3) What are the factors affecting the level of success among EDs? Lastly, working in collaboration with the BC Ministry of Health as our policy-maker partner, the research will investigate how the demonstrated efficacy of this research can be applied on a provincial and national level to establish a template for policy makers from other jurisdictions to translate knowledge into action for EDs. This research study will employ the IHI model for improvement, incorporate the principles of participatory action research, and use the E2E online CoP to engage ED practitioners (eg, physicians, nurses, and administrators, exchanging ideas, engaging in discussions, sharing resources, and amalgamating knowledge) from across BC to (1) share the evidence of early intervention in sepsis, (2) adapt the evidence to their patterns of practice, (3) develop a common set of orders for implementing the sepsis pathway, and (4) agree on common indicators to measure clinical outcomes. Our hypothesis is that combining the social networking ability of an electronic CoP and its inherent knowledge translation capacity with the structured project management of the IHI model will result in widespread and sustained improvement in the emergency and overall care of patients with severe sepsis presenting to EDs throughout BC.

## Introduction

### Project Overview

Recent medical research literature on emergency sepsis management demonstrates that early goal-directed therapy (EGDT), composed of the rapid implementation of a sequence of diagnostic and management steps by a cohesive, interprofessional team in the emergency department, can positively influence morbidity and mortality rates. One urban emergency department in British Columbia (BC), Canada, has successfully integrated the EGDT protocol into the emergency care map of septic patients and demonstrated significant improvement of septic patients’ outcomes. A care map can be viewed as a workflow diagram using basic flowchart symbols, such as process block, decision, and document, to map out and describe the details of the care process. Disseminating and diffusing this team-based practice throughout the rural and urban emergency departments in BC is integral to significantly reducing morbidity and mortality provincially.

The practice improvement model advocated by the Institute for Healthcare Improvement (IHI) is recognized as an effective strategy to assist teams and organizations to implement system improvement processes. In addition, a community of practice (CoP) is touted as a useful social networking model to aid health care teams toward mutual learning and knowledge exchange. Meanwhile, modern information technologies connected through the Internet are revolutionizing how individuals and groups from disparate geographic locations can effectively communicate and collaborate together. The opportunity is ripe in health care to examine and rigorously evaluate how information technologies can facilitate the construction and nurturing of an electronic community of practice (eCoP) to support the implementation of a quality improvement model (informed by the IHI practice improvement model) to harmonize team-based health practices.

#### Study Objectives

What are the impacts of engagement in the sepsis quality improvement collaborative on uptake of and adherence to evidence-based sepsis guidelines and the management of sepsis in the context of emergency medicine? A collaborative, as defined by the IHI, is a time-limited effort (usually 9–18 months) of multiple organizations that come together to learn about and to create improved processes in a specific topic area. The expectation is that the teams share expertise and data with each other; thus, “Everyone learns, everyone teaches.”

Specific research questions include (1) How is the quality improvement collaborative implemented, and how do members within and across emergency department sites engage in the collaborative? What are participants’ perceptions, experiences, and satisfaction levels related to engaging in the collaborative processes? (2) What are the practice patterns of sepsis management within and across emergency department sites? Have the patterns of sepsis care at emergency department sites changed over time? To what degree have patterns been harmonized across the emergency department sites? and (3) What change has occurred in clinical sepsis data collected over time within and across emergency department sites? What are the contextual factors that influence clinical impact?

#### Study Design and Evaluation

The University of British Columbia Faculty of Medicine, in collaboration with the BC Ministry of Health, the six BC Health Authorities, and emergency departments throughout BC conducted a needs assessment of emergency departments across BC in 2007 and formed a trial collaborative in 2008 under the Evidence to Excellence Initiative, referred to as the E2E pilot. This current research will build on the work of the E2E pilot to engage 18 emergency department teams from across BC to form a quality improvement sepsis management collaborative supported by an eCoP, to implement the sepsis EGDT guidelines during a 3-year period. Mixed method evaluation will include (1) tracking five clinical outcome indicators in each participating emergency department, (2) understanding health professionals’ and administrators’ perspectives, and (3) evaluating the development, implementation, and change of each emergency department’s clinical care mapping. This is a timely and novel area of research, as there are no published studies demonstrating and documenting the combination of a quality improvement collaborative with an eCoP to support a community-building approach toward systemwide practice coordination of sepsis management in emergency departments.

### Background

This section highlights three key areas salient to our research proposal. First, we discuss the scope of the problem and evidence-based management of patients with sepsis in emergency departments. Second, we highlight the knowledge translation challenge of systematically implementing evidence-based strategies into emergency departments in BC. Third, to meet the provincial objective of enhancing sepsis management to optimize knowledge translation, we explore the literature on the IHI collaborative process in quality improvement implementation, and the literature on CoP and eCoP in enhancing team communication and knowledge exchange. Fourth, with some existing examples to illustrate the application of an eCoP in practice, we propose combining a quality improvement model with an eCoP as the theoretical basis of our study proposal.

#### Sepsis Management in the Emergency Department

Sepsis is a complex syndrome comprising a range of clinical conditions caused by the body’s response to an infection, which can develop into severe sepsis resulting in organ failure and death. Severe sepsis is a major and underappreciated cause of morbidity and mortality worldwide. There are 750,000 cases of severe sepsis annually in North America, and approximately 1400 people worldwide die daily of sepsis [1,2]. Due to its aggressive and multifactorial nature, of those who die of sepsis, about 30% die within a month of diagnosis and about 50% within 6 months [3,4]. In addition to being a leading cause of intensive care unit admissions, sepsis consumes tremendous emergency department resources. Data indicate that suspected sepsis accounts for 500,000 emergency department visits annually in the United States, and that these patients spend an average of almost 5 hours in the emergency department [5].

Recent years have seen a revolution in the diagnosis and treatment of sepsis, starting with the development of operational definitions for severe sepsis and septic shock [6]. Increasing evidence demonstrates that timely delivery of appropriate therapy significantly improves outcomes in patients who have severe sepsis [7]. Such therapies include the rapid delivery of appropriate antibiotics and aggressive resuscitation [3]. Kumar et al demonstrated an average 7.6% decrease in survival for each hour’s delay in giving antibiotics from the time of onset of hypotension (shock) [8].

In 2004, the Surviving Sepsis Campaign brought together critical care and infectious disease experts representing 11 organizations, including the Society of Critical Care Medicine and the European Society of Intensive Care Medicine. The work of this campaign resulted in the development of guidelines for the early and comprehensive management of severe sepsis, known as EGDT, with the aim of reducing mortality by 25% within 5 years [7]. Key components of this strategy include aggressive fluid administration, early antibiotic usage, use of various physiologic measurements to guide the resuscitation of the acutely septic patient, and prompt admission of patients to close monitoring and care such as in intensive care units. The EGDT guidelines were endorsed by the IHI, who translated them into resuscitation bundles, or grouped interventions. These guidelines were updated in 2008 [9].

One example of success achieved with these guidelines in BC has been the experience at St Paul’s Hospital, a 500-bed tertiary care academic hospital in Vancouver, with a 15-bed intensive care unit and an emergency department that sees over 60,000 patients per year. The implementation of the EGDT guidelines included a set of early identification indicators, a computerized physician order entry system set for suspected sepsis, the introduction of invasive hemodynamic monitoring, and a sepsis kit in the emergency department. By converting the evidence from the guidelines into a clear algorithm, raising awareness of sepsis with an extensive education campaign, and integrating several key interventions into the emergency care map of septic patients, they achieved their targets of clinically significant improvements in process outcomes and in the survival of patients who presented to the emergency department because of severe sepsis. In the 6 months following introduction of the guidelines, improvements were observed in time to initiation of EGDT (3.2 vs 10.4 hours, *P *= .001) and to achievement of resuscitation goals (10.4 vs 30.1 hours, *P *= .007), as well as a trend toward more rapid antibiotics administration (1.4 vs 2.7 hours, *P *= .06). This was associated with a decrease in hospital mortality rate to 27.0% from 51.4% in the preprotocol patients (absolute risk reduction = 24%; 95% confidence interval 3%–37%). Improvements in process measures and clinical outcomes, including mortality, were sustained at 16 months after implementation.

#### Challenge of Systemwide Knowledge Translation of EGDT in BC

Implementation of evidence-based guidelines and protocols into a health system with multiple health teams is a recognized and significant challenge [10,11]. It is clear that simply disseminating the latest evidence to health professionals through conferences and other traditional educational settings lacks the ability to influence individual health professionals or health system change. Clearly, to effectively incorporate evidence-based guidelines for patient care management in the emergency department, much work needs to be done beyond making the team aware of such protocols.

For health care teams and their members in the health system who are actively seeking practice improvement together, knowledge translation requires intervention beginning with the individual and progressing through to health care teams in the system. Acquisition of necessary knowledge and skills by individual health professionals needs to be synchronized with the establishment of quality improvement strategies to implement change and measure outcome by the entire team. In addition, a mechanism of knowledge exchange between teams in the health system is necessary to spur mutual learning and mentoring. Therefore, three key aspects are needed to bring about an effective and lasting change: (1) an evidence-based approach to guide clinical practice, (2) an engaged interprofessional team of emergency health care providers, and (3) an effective implementation and quality improvement process to engineer the redesign of clinical pathways among different teams to work together in a health system.

In the case of sepsis management in BC, as earlier stated, the emergency department team at St Paul’s Hospital has successfully provided pilot evidence of the benefits of EGDT. How can we now help spread this practice throughout emergency departments in BC to magnify these health outcome benefits provincially? How should the variations of the different emergency departments (eg, urban, regional, and rural practice environments) with variability in resources availability (eg, laboratory testing, access to intensive care units) be appropriately accounted for in EGDT implementation? How do we promote mutual learning and knowledge exchange among these different emergency department teams? How do we facilitate the establishment of provincewide quality improvement and evaluation mechanisms for measuring change? BC will need a three-pronged strategy to (1) assist individual emergency department health professionals in knowledge acquisition and behavioral change based on this evidence-based approach of EGDT, (2) establish a provincial implementation and quality improvement process with buy-in from all participating emergency departments to share the joint vision to improve provincial sepsis outcomes, and (3) engage the rural, regional, and urban emergency department teams in BC to establish their own team approach for implementing EGDT based on best evidence, available resources, and their unique contexts to animate the provincial quality improvement process.

While there is no proven, unified model in the literature to achieve all three prongs of the strategy, the literature offers three separate approaches, each specifically fulfilling an important aspect of the overall knowledge translation strategy. They are (1) the collaborative quality improvement model advocated by the IHI for establishing a quality improvement initiative, (2) the CoP approach in building a community of health professionals, and (3) the use of information technologies to capture knowledge and promote communication that overcomes geographic barriers. Each of these three approaches is highlighted in detail below.

#### The Quality Improvement Collaborative Model

The proposed research incorporates the IHI’s quality improvement collaborative model [12]. Recall that a collaborative is a time-limited, structured improvement project. Teams participate in a series of sessions where they learn how to plan, implement, and measure the impact of changes intended to create improvement. Between sessions, ideas are incrementally applied and tested locally. Improvement teams also share with other teams what has and has not worked for them.

Several health care organizations have seen breakthrough improvements in quality while reducing costs by adopting the IHI collaborative improvement model. Between 1995 and 2003, IHI sponsored over 50 collaborative projects. The results of these projects were dramatic and included reducing waiting times by 50%, reducing worker absenteeism by 25%, reduced intensive care unit costs by 25%, and reduced hospitalizations for patients with congestive heart failure by 50% [11].

#### Communities of Practice in Health Care and Emergency Medicine

Etienne Wenger, the acknowledged pioneer of the term community of practice [13], defined it as: “…groups of people who share a concern, a set of problems, or a passion about a topic, and who deepen their knowledge and expertise in this area by interacting on an ongoing basis” [13]. Key aspects of CoPs are (1) defining the scope of the community, (2) engaging committed participants, (3) identifying common needs, (4) outlining the goals or terms of reference of the community, (5) maintaining members’ interest and involvement, (6) growing the community, (7) developing a knowledge base, and (8) adding value [13]. This CoP concept was supported and applied in organizational behavior in business [14], and has also been shown to be an effective means for promoting evidence-based practice, patient-centered collaborative care, knowledge sharing and creation, and emotional or collegial support [15]. The resultant infrastructure allows sharing of clinical and operational practices, and allows that information to be contextualized and adapted to the local medical environment, thereby improving uptake. We see this to be an effective social networking environment to promote collaboration for health practitioners and administrators working in BC emergency departments. Health practice is more than an individual pursuit, and the learning process has to be seen, conceptualized, and realized as an iterative process.

#### The Use of Information Technologies to Enhance Knowledge Translation

Information technology enhances the capabilities of a CoP [16]. An online emergency medicine CoP can provide members with the opportunity for peer-to-peer learning and problem solving from anywhere at any time. This can help to overcome the challenges of geography and time that face emergency medicine health care providers. Several empirical studies have shown the benefits an eCoP can have within the health care sector generally [17-20], and in the context of emergency medicine specifically [21,22]. We intend to build on past sepsis management research by purposefully embedding information technologies into the CoP and measuring its utility in enhancing communication and knowledge sharing. In addition, we intend to demonstrate process improvement and subjective perception of learning of health professionals, as well as the progressive harmonization of practice patterns between emergency departments throughout the Sepsis Quality Improvement Collaborative.

#### Context: Evidence to Excellence Pilot

In 2007, the BC Ministry of Health provided funding to the E2E pilot and conducted a needs assessment with emergency health professionals to better understand their needs in optimizing the quality improvement of clinical areas and operational processes in emergency services delivery. This assessment revealed the necessity to share resources, have access to the latest evidence, network with their peers, have a means to implement the latest evidence, and have access to assistance to facilitate quality improvement implementation.

In 2008, the E2E pilot ran a trial collaborative on the clinical topic of sepsis and the operational topic of triage. The committees selected these topics during the needs assessment. The E2E pilot’s trial collaborative has recruited 55 teams (18 sepsis collaborative teams and 27 triage collaborative teams) from 31 emergency department sites across BC. There were 170 participants across these 55 teams working to improve sepsis care and triage processes at their sites through the IHI collaborative process that is administered by the E2E pilot. This operational pilot provided an understanding of the mechanics of running a collaborative; however, no evaluation was completed for the trial collaborative.

#### Electronic Community of Practice

The E2E pilot also built a trial beta version of an eCoP environment for the trial collaborative. This included an overarching E2E eCoP, which allowed all practicing BC emergency medicine professionals to share resources, participate in discussion forums, and view relevant upcoming events. The eCoP’s purpose is to build a virtual CoP by providing an online (Web-based) space to exchange ideas and discussions, share resources, and amalgamate knowledge. In the context of E2E, the eCoP will build this virtual community for professional (clinicians and administrators) working in emergency departments across BC.

The key components of the eCoP are *resources*, *discussion forums*, *events*, *WikiDocuments*, and *members*. The resources component provides the ability to post, view, and download resources or files that would be beneficial to personnel working in emergency departments. The resources are organized in folders. The discussion forum component provides threaded discussion forums. The eCoP can accommodate as many forums as the community needs, which are organized by folder and then by the topic. The events component allows moderators and organizers to add events to a calendar function that are of interest to the community, which can be viewed by all members. The WikiDocuments component provides a working document where multiple people can collaboratively develop a body of work, which can be viewed by all members of the community. Finally, the members component allows members to search for their colleagues and have access to the contact information that each member has granted permission to be shared by the community.

The architecture for access to the eCoP for the E2E pilot is described below. The main portal to the eCoP would be from the E2E website. Once first-time users access the eCoP, they can sign up for immediate access by entering their profile information, and returning users will simply log in. The E2E eCoP will be open to all interested people, and there will be no approval process for access. This E2E eCoP will be an exchange for all areas relating to emergency medicine, including clinical topics, operational topics, and improvement methods or strategies. This overarching E2E eCoP will provide access to the current time-limited, project-based eCoPs that E2E is facilitating, such as the sepsis collaborative, which is relevant to this proposal. The sepsis eCoP (and other topic eCoPs) will be restricted to those people who are participating in the sepsis improvement project to provide a safe collaborative space to share ideas and overcome barriers to improvement. No log-in will be necessary; however, users will need approval prior to access. Each eCoP will have all the key components previously described.

Additionally, there was an eCoP for each active trial collaborative, which provided a discussion around improvements and best practices, and provided a forum to post data and change concepts. This beta eCoP, with active membership of approximately 160 and started on May 15, 2007, had 637 visits and over 12,000 pages viewed since inception. The unprecedented opportunity to carry out a rigorous evaluation of this eCoP in its contribution to interprofessional learning, knowledge exchange and capturing, and contribution to quality improvement in provincial sepsis management will be both highly relevant and timely.

## Methods

The overarching purpose of the proposed research is to implement a *quality improvement collaborative *and evaluate how emergency departments across BC engage in this model to make improvements in *sepsis management*. We use the IHI definition of a collaborative: “a time-limited effort (usually 9–18 months) of multiple organizations that come together to learn about and create improvement processes in a specific topic area” [12] through sharing of knowledge, experiences, and data. We will evaluate the processes of collaboration and harmonization of sepsis management within and across emergency department sites engaging in the collaborative.

This research study will employ a multiple case studies approach and will use both qualitative and quantitative methods of analysis. It will incorporate the principles of participatory action research and use an eCoP to engage emergency department practitioners (eg, physicians, nurses, and administrators) from across BC. Specific outcomes of this study include (1) sharing the evidence of early intervention in sepsis (eg, exchanging ideas, engaging in discussions, sharing resources, and amalgamating knowledge), (2) adapting the evidence to their patterns of practice, and (3) developing a common set of guidelines or protocols for implementation of the sepsis pathway.

### Research Questions

The principal research question for the proposed study is as follows: What are the impacts of the sepsis quality improvement collaborative on uptake of and adherence to evidence-based sepsis guidelines and the management of sepsis in the context of emergency medicine?

This will be addressed through the following specific research questions:

1. How is the quality improvement collaborative implemented, and how do members within and across emergency department sites engage in the collaborative? What are participants’ perceptions, experiences, and satisfaction levels related to engaging in the collaborative processes?

2. What are the practice patterns or care maps of sepsis management within and across emergency department sites? Have the patterns of sepsis care at emergency department sites changed over time? And to what degree have patterns harmonized across the emergency department sites?

3. What change has occurred in clinical sepsis data collected over time within and across emergency department sites? What are the contextual factors that influence clinical impact?

4. In what ways has the eCoP contributed to the change in individual health professionals’ attitude, knowledge, and skills, and in the practice patterns and septic patient care maps of the emergency departments involved?

### Overview of Procedures

Evaluation will use a participatory action approach. Throughout the course of this project, we will evaluate the collaborative and collect case study data including care map data and clinical outcome data. Table 1 highlights some of the main activities during the 3-year project.

**Table 1:** Main activities of the 3-year sepsis quality improvement collaborative project.

Year	Objective	Associated activities
1	Synthesize E2E^a ^pilot data	Synthesize the E2E pilot data and its trial collaborative to inform the current project and best prepare for the sepsis collaborative in year 2.
	Develop data collection system and infrastructure	Develop an online data collection system and a strategy for uniform collection of clinical sepsis data. This includes training and recruitment of personnel in central data collection.
	Complete prework for sepsis collaborative	Recruit emergency department sites and teams to engage in the sepsis collaborative and associated case study research.
2	Develop baseline care maps for each recruited site	Systematically and uniformly create baseline care maps for each participating site to outline current sepsis management care processes.
	Establish a common quality improvement framework	At the beginning of year 2, hold a common session to look at evidence of early goal-directed therapy, jointly examine the protocol, and agree through a consensus-building process on a set of five core indicators of measurement to be collected (see Clinical Sepsis Data in the Measures section) to facilitate cross-site comparisons.
	Run the sepsis collaborative; conduct three sepsis learning sessions for emergency department personnel	(1) Hold learning session 1 in May 2010, with first action period from May to September 2010, (2) hold learning session 2 in September 2010, with action period from September 2010 to January 2011, (3) hold learning session 3 in January 2011, with final action period from January 2011 to March 2011.
	Develop postcollaborative care map	Using the same methods for developing the baseline care map, create postcollaborative care map.
3	Evaluate sepsis collaborative	Analyze findings, conduct participant focus groups and survey, and evaluate sepsis indicators from data that were collected.
	Evaluate sustainability and dissemination of sepsis improvements	Evaluate the success and barriers to the sustainability and translation of the sepsis improvements that have been realized through the collaborative.
	Develop sustainability and dissemination strategy	Develop a strategy to sustain gains in sepsis management and a strategy to disseminate best-care practices to other sites across British Columbia.

^a ^Evidence to Excellence.

### Design

The proposed research uses a multiple case studies design, which relies on both qualitative and quantitative data and analysis. The 18 sites engaged in the pilot will be approached and recruited for the proposed research. Multiple data sources will be documented and examined to effectively “tell the stories” of the 18 cases in terms of practice, engagement in the collaborative, and quality improvement in sepsis care.

Community engagement will be an iterative process that spans the initiative (including planning), implementation, training, evaluation, and future direction settings (eg, sustainability). Ethical approval to conduct this proposed research will be sought whenever and wherever necessary. The project team will establish and work according to the guidance of an advisory committee comprising collaborative members and health professionals. Research is intended to engage individuals in the emergency department sites (including both health professionals and health administrators of the department, or the hospital or health authority) to be part of the evaluation team and develop research and evaluation skills. Finally, to engage the participating emergency department sites in all aspects of the study, the research framework will be flexible and responsive to member input.

### Settings and Participants

We define a *case *as a participating emergency department site. The care map related to sepsis management will be a core aspect of the case study. For this proposed research, *care mapping *can be viewed as a workflow diagram using basic flowchart symbols, such as *process *block, *decision*, and *document*, to map out and describe the details of the care process. The collaborative or team members represent the individual participants. The current trial collaborative, facilitated by the E2E pilot, has provided an understanding that the participants agree to collect clinical data and are willing to volunteer their time to this work to improve sepsis management. Because each emergency department site is resourced differently, different clinical sepsis data are collected at each site. For the proposed research, core sepsis data available across sites will be examined.

### Measures

The proposed research will identify and describe each case, collect baseline and comparison data, gather participant perception data, monitor practice changes, and track clinical sepsis measures.

As earlier stated, multiple case studies will be used to document practice and engagement in the collaborative at individual sites, as well as to understand the interaction within and across sites in the context of the sepsis collaborative. In systematically describing the cases, we will gather information related to the participants, workflow, and sepsis management at the emergency department and artifacts created through engagement in the sepsis collaborative. These include usage data from the eCoP platform, perceptions and experiences, and documents created by participants as they engage in quality improvement projects in the sepsis collaborative.

Understanding the relationships and interactions within, among, and between the case sites will provide the basis for documenting and evaluating how change occurs. Clinical sepsis data will be used to understand the improvements that potentially result from engaging in the collaborative. We will include three main areas of measurement, as follows.

#### Perception and Experience Data

We will use a variety of qualitative and quantitative measures to evaluate and understand the participants’ perspectives on the following: implementation of protocols; participant engagement and collaboration through the use of the eCoP environment; collaboration within and across emergency department sites; and implementation and use of the IHI collaborative model within and across sites throughout the project. This will enable a foundational understanding of the processes that emerge. We aim to understand differences and similarities across sites, as well as how the eCoP is used and contributes to collaboration processes. Data collection will include focus groups of health care practitioners at the emergency department sites and individual surveys (3–20 participants/site). eCoP usage data will be collected to understand level of engagement and processes of collaboration, thus providing recommendations for future use in the collaborative. The usage data will include the following: (1) data on the number of visits to the eCoP website, which can be distilled to monthly, weekly, and daily, as well as the location of the visiting member, (2) data on the average time each user spent on the website, and (3) the number of pages viewed, which will indicate which resources, discussion forums, and calendar events were most popular.

We will interview a cross-section (multiple sites with various health professionals) of collaborative participants. Interviews will capture pre- and postinvolvement expectations, experiences engaging in the collaborative, and perceptions of impact of engagement in the collaborative on sepsis management. This will constitute a formative evaluation of participant engagement. Online focus groups will be carried out throughout the project, within the eCoP’s discussion boards. Data captured from the eCoP (eg, discussions and downloaded resources) will be examined. Online surveys will be used to assess participant satisfaction and provide feedback at regular intervals.

At the end of year 3, we will conduct focus groups of emergency department site participants to gain postcollaborative (end point) perspectives. Participants will be asked to reflect on future sustainability of the collaborative and sepsis improvement processes. These same issues will be addressed in detail through interviews with a subsample of stakeholders and participants.

#### Practice Change Data

Another important component of the proposed research is to evaluate the extent to which the various participating emergency departments across the province become harmonized in their respective management of patients with sepsis. Central to the ability to evaluate the degree of harmonization is care mapping. Each emergency department site will create an initial (baseline) care map (ie, a care map of how sepsis patients are, on paper, to be managed) and will be asked to develop subsequent care maps at the end of each year during the 3-year proposed research to accurately reflect current practice in sepsis management. Early on, participating sites will be invited to participate in a learning session related to care mapping led by collaborative member(s) with expertise in care mapping processes.

We recognize there is a potential for the Hawthorne effect—a change in behavior or performance due to a change in environmental conditions—to take place; however, an asset of the 3-year duration of this research is that the potential impact of the Hawthorne effect is reduced with the reinforcement of improved practices over time. Further, engagement in the collaborative will provide opportunities for participants to systematically reflect on their practices in the course of the learning sessions, and ongoing discussion of best practices with colleagues and experts.

While the concept of care mapping is not new, the unique aspect of our proposed research is that funding from the Canadian Institutes of Health Research will enable us to develop a quantifiable index that highlights the harmonization of sepsis management across emergency department sites in BC to compare the degree of similarity of care maps across the province and the change in similarity (either more or less similar) over the 3-year time frame. Calculating this index would be similar to the bioinformatics’ approach in calculating the similarity of genes between different people with similar illnesses, thereby locating the responsible segments of the genes that lead to the expression of the disease. Furthermore, the process of evaluating and analyzing the adherence to care maps and successes and challenges may eventually lead to a context-specific clustering of care maps for emergency departments in rural, regional, urban, and provincial contexts. This approach can set a benchmark for future endeavors in refining the application of protocols in different contexts.

#### Clinical Sepsis Data

The evidence-based literature shows that several clinical measures are important in the management of sepsis. Recognizing that emergency department sites across the province have varying resource capacities and to prevent the collection of clinical sepsis data becoming too cumbersome for the participating emergency department sites, five *core *measures will be collected for this proposed research: (1) time to administration of antibiotics, (2) time to doing blood cultures before antibiotics are administered, (3) emergency department length of stay, (4) length of time between the patient’s arrival and when the provincial bed management transfer call is made (to facilitate the transfer of patients to a facility with intensive care capabilities or contacting a consultant for admission and definitive care) or admission to the hospital’s own intensive care unit, and (5) mortality within 2 months, the cause of death, and its relationship with the sepsis episodes. These measures have been found to be practical and feasible to collect at each of the sites.

Members of the E2E pilot have demonstrated their willingness to collect clinical sepsis data in addition to their day-to-day workflow. Relying solely on nurses’ and physicians’ notes as the primary source of recorded data can be challenging; therefore, we will develop a formalized structure for recording the data, including a 1-page data form with all the core indicators (clinical sepsis data) that can be filled out in real time and faxed to a central location for collection. This form will also be able to be filled out digitally and sent through the eCoP to a central location for collection. Therefore, the eCoP can also become a medium for emergency department practitioners to upload their data in a secure and user-friendly manner that enhances the degree to which clinical sepsis data are collected.

The proposed research would contribute to the realization of various benefits for participating emergency department sites. Engaging in the collaborative and the evaluation will enhance awareness of sepsis management and create a more streamlined implementation of treatment for septic patients. Further, the proposed research will enhance the capacity of each emergency department to collect measurement data, using local knowledge to improve local systems.

### Analysis

The complexity of the natural environment necessitates a multiple case study model. Content analysis using the constant comparative method will be used to analyze the survey, focus group, and interview data. Thematic analysis will be used to understand participants’ perceptions, satisfaction, practice patterns, and collaborative engagement patterns. Some quantitative data will also be collected for technical usage statistics. For example, the eCoP will be set up to track what information was accessed, for how long, and in what sequence. This will be an anonymous process and will be used simply to understand how users interact with the content (not a surveillance of individual usage patterns). The five commonly agreed-on process and clinical sepsis indicator data will be compared within sites across time from baseline, at midpoint, and postintervention. We will use these data to analyze the influence of eCoP on practice and outcome change by seeing which group(s) improved and when, and how these improvements correlate with the qualitative data from eCoP.

Care maps will be analyzed and compared within and across sites to document evolutions in their approximation to the clinical practice guidelines, similarities and differences between different care maps, and changes in these care maps over time. Clinical care maps will also be analyzed based on their geographic locations to see whether there might be more similarities between rural, regional, or urban locations.

We will compare clinical sepsis data within and across sites to capture benefits to sepsis management. By looking across sites, we may identify certain processes that connect with clinical outcomes and further disseminate that information along with associated contextual factors across sites.

## Contingency Planning

At this phase in our research, we anticipate that all the aspects of this proposal can be reasonably achieved. However, we foresee potential issues and difficulties that may affect the scope of our research. We also propose strategies to alleviate the foreseeable challenges that we may encounter during this proposed research.

### Data Collection Rules and Regulations

Various BC health authorities may have differing rules and regulations governing the release of patient data. Before beginning data collection, we will consult the respective risk management or information services department for each health authority to ensure that we can obtain the appropriate permission to collect the required sepsis indicator data.

If receiving permission to collect sepsis indicator data poses a barrier, we will consult with the existing E2E pilot to examine the data that have already been collected as part of its sepsis collaborative for use in our proposed research.

The challenge is that the data that have been and are being collected by the E2E pilot may not be as comprehensive as what we require within the scope of our research.

### Data Collection Capacity of Emergency Departments

Members of each emergency department’s team participating in this research will be required to record and collect specified indicator data. This will be in addition to their existing clinical workload. Team members may not be able to fully capture the required indicator data because of their current workloads. If this is the case, additional resources may be put in place to provide emergency department sites with dedicated staff to collect necessary indicator data. These dedicated staff may take the form of a research assistant designated to collect and report the indicator data at respective sites.

### Quality of Data Collection

In addition to the resource capacity of various emergency department sites participating in this research, emergency departments will have varying levels of experience in implementing sepsis protocols. Some emergency department teams may have already been implementing a sepsis protocol for a few years, while it may be the first time for other sites. As such, the experienced emergency department teams may be able to provide very rich indicator data, while the teams implementing a sepsis protocol for the first time may be able to provide a high level of basic data only in the short term.

## Committees Guiding This Initiative

We will establish an advisory committee to guide our project development, implementation, and evaluation over the 3-year project and ensure it aligns with the development of the provincial health system. Representatives on this committee will include a senior policy maker from BC Ministry of Health, a senior policy maker in one of the health authorities, the chair of E2E committee, the Department Head of Emergency Medicine from the Faculty of Medicine, University of British Columbia, an expert in the area of CoP and related research, and an information technology expert. This committee, with a maximum of 10 people including the principal investigators and co-investigators of this project, will act as a sounding board to explore interface issues between the health authorities and Ministry of Health, the health professionals, the provincial emergency institutions, and the patients. This committee will also provide feedback on progress, give advice regarding the iterative harmonization of this initiative with the provincial emergency medicine landscape, and engage within their own organizations and others for future knowledge translation and sustainability of our initiative beyond the project period. The Australian representative, with strong experience in stimulating collaboration among emergency departments in Victoria, will provide great insights into our efforts to ensure ongoing success [21]. Further, since September 2007, the E2E pilot established a provincial working committee, composed of emergency physicians, nurses, and emergency department and health authority administrators, supported by funding from the Ministry of Health. This group will form a common vision of how to use an eCoP and the IHI collaborative model to improve emergency care. The working group, with relevant and solid clinical experiences in applying health evidence to emergency care practices, will provide the invaluable opportunity to support and rigorously evaluate the efficacy of and gain invaluable insights into this IHI-eCoP approach in the provincial implementation of clinical evidence into practice.

## Dissemination of Findings and Translation of New Knowledge

We expect that this project will ascertain the role of an eCoP in the IHI change management process in harmonizing provincial evidence-based medicine in the emergency medicine community. The close working relationship with policy makers through their project involvement and the advisory committee will ensure that health professionals, policy makers, health administrators, and researchers work synergistically toward systems improvement, helping to achieve true knowledge translation and using evidence to guide the evolution of the health system. The relationship foundation established through this project will be important for not only the sepsis protocol, but also future clinical practices in emergency medicine.

In addition, we will share the findings of the proposed research with the professional community through the following. (1) We will submit our results for publication in peer-reviewed journals and present abstracts at professional conferences. We will emphasize effective eCoP approaches in implementing clinical practice guidelines by emergency health professionals through the IHI process of practice improvement. (2) We will collaborate with the BC Ministry of Health and health authorities to ensure that lessons learned from implementing sepsis guidelines using the eCoP-supported IHI process in this initiative will be translated into future clinical pathways for health professionals. (3) We will distribute reports of the findings to organizations whose primary focus is, and whose membership has interest in, knowledge translation and patient safety, such as the Canadian Patient Safety Institute and the Canadian Health Services Research Foundation, or provincial organizations such as the Michael Smith Foundation for Health Research. (4) We will collaborate with the BC Medical Association, Association of Registered Nurses of BC, and the University of British Columbia’s Faculty of Medicine, Department of Emergency Medicine in devising ways to effectively promote eCoP to their respective membership. (5) We will disseminate the eCoP-supported IHI process to physicians, nurses, and other allied health professionals in BC and beyond through continuing medical education conferences and workshops to influence health professionals’ behavioral change in evidence-based health practices through interprofessional learning and team-based practices. (6) We will raise awareness of eCoP to promote intercollegial communication so that it becomes an integral component of patient safety and evidence-based medicine in health care delivery and in quality improvement. (7) We will develop and implement an educational program to help health practitioners use eCoP and IHI processes effectively, tailoring this program to the needs of health professionals (eg, physicians and nurses) in practice and in training, as well as the needs of interdisciplinary teams of health care providers.

## Significance

Overall, the findings of this project will contribute to the overall goal of the provincial initiative to increase care harmonization and incorporation of clinical evidence into routine practices in the emergency medicine environment through eCoP-supported IHI process. Further, this research proposal is significant for several key reasons. First, this is a novel area of research because there are no published studies combining CoP and information technologies to promote community building and the IHI improvement process supported by this community-building approach. Second, it will provide an understanding of how to best facilitate uptake of evidence-based health practices and education into the workflow of health professionals in the emergency department throughout the province to maximize utility and engagement. Third, it will help us understand how best to improve patient safety through the accurate recognition and management of acute sepsis. Fourth, it will benefit many groups in the following manner: (1) physicians, nurses, and other health professionals in emergency medicine will have useable and convenient online tools to assist them in providing better patient care and patient self-management, (2) health professionals will enhance their skills, (3) patients will benefit from the accurate identification of sepsis by emergency health personnel, and (4) communication among all three CoPs—health professionals, health administrators, and policy makers—will be facilitated and sustained. Our study will help health care professionals to maximize their skills, will ensure that technology is used appropriately, and will work together with health administrators and policy makers to ensure harmonization of best practices as we know them today, and an optimal provincial networking structure to support evidence-based health practices tomorrow. Finally, this study will help to develop the ability to measure harmonization of clinical practices using the clinical care map method and to elucidate how we might be able to look at the variation in care map evolution due to geographic differences, based on research evidence that often gets synthesized into only one set of clinical practice guidelines for urban practices. This will help professionals in the health care system to not only understand tangibly but also support the optimal care models.

## Abbreviations

BC: British Columbia
CoP: community of practice
E2E: Evidence to Excellence
eCoP: electronic community of practice
ED: emergency department
EGDT: early goal-directed therapy
IHI: Institute for Healthcare Improvement
